# Risk Factors for Seizures Among Young Children Monitored With Continuous Electroencephalography in Intensive Care Unit: A Retrospective Study

**DOI:** 10.3389/fped.2018.00303

**Published:** 2018-10-15

**Authors:** Jan Vlachy, Mingyoung Jo, Qing Li, Turgay Ayer, Pinar Keskinocak, Julie Swann, Larry Olson, Atul Vats

**Affiliations:** ^1^School of Industrial and Systems Engineering, Georgia Institute of Technology, Atlanta, GA, United States; ^2^Children's Healthcare of Atlanta, Atlanta, GA, United States; ^3^Johns Hopkins Applied Physics Lab, Baltimore, MD, United States; ^4^Department of Industrial and Systems Engineering, North Carolina State University, Raleigh, NC, United States; ^5^Departments of Pediatrics and Neurology, Emory University, Atlanta, GA, United States; ^6^Department of Pediatrics, Emory University, Atlanta, GA, United States

**Keywords:** continuous EEG, pediatric, non-convulsive, intensive care, brain injury

## Abstract

**Objective:** cEEG is an emerging technology for which there are no clear guidelines for patient selection or length of monitoring. The purpose of this study was to identify subgroups of pediatric patients with high incidence of seizures.

**Study Design:** We conducted a retrospective study on 517 children monitored by cEEG in the intensive care unit (ICU) of a children's hospital. The children were stratified using an age threshold selection method. Using regression modeling, we analyzed significant risk factors for increased seizure risk in younger and older children. Using two alternative correction procedures, we also considered a relevant comparison group to mitigate selection bias and to provide a perspective for our findings.

**Results:** We discovered an approximate risk threshold of 14 months: below this threshold, the seizure risk increases dramatically. The older children had an overall seizure rate of 18%, and previous seizures were the only significant risk factor. In contrast, the younger children had an overall seizure rate of 45%, and the seizures were significantly associated with hypoxic-ischemic encephalopathy (HIE; *p* = 0.007), intracranial hemorrhage (ICH; *p* = 0.005), and central nervous system (CNS) infection (*p* = 0.02). Children with HIE, ICH, or CNS infection accounted for 61% of all seizure patients diagnosed through cEEG under 14 months.

**Conclusions:** An extremely high incidence of seizures prevails among critically ill children under 14 months, particularly those with HIE, ICH, or CNS infection.

## Introduction

Nonconvulsive seizures in intensive care unit (ICU) patients do not manifest convulsive activity, but they can be detected through continuous electroencephalography (cEEG) (1, 2). These seizures are more prevalent among critically-ill children than previously thought (2). Furthermore, growing evidence suggests that non-convulsive seizures in ICU are associated with worse patient outcomes (3–5). To detect non-convulsive seizures, monitoring patients at high seizure risk with cEEG is crucial. However, interpreting cEEG is very labor intensive and requires substantial investment in equipment, technician support, and network infrastructure (5). Therefore, it is not feasible to universally monitor all ICU children with neurological conditions, which motivated us to systematically identify high-risk conditions that warrant monitoring.

Certain groups of children have already emerged as having a higher risk of seizures in several studies (2, 6–12); however, these findings remain limited because of small sample sizes. A multicenter study (13) attempted to address sample size, however, it included few young children and children with acute structural diagnoses. There is a consensus statement for monitoring children, a guideline for neonates (5, 14) and a risk prediction model for children (15). Our objective in this large single center study is to identify high-risk conditions in a large cohort of young children.

## Materials and methods

### Setting

The retrospectively-reviewed study population consisted of all children who underwent cEEG monitoring at the Egleston campus of Children's Healthcare of Atlanta (Children's) from June 2010 to August 2013.

The Egleston campus of Children's is a 255-bed free-standing children's hospital providing quaternary care. For these children, the attending physician is responsible for ordering monitoring. In the hospital, cEEG monitoring occurs in the following units: a 30-bed medical-surgical pediatric intensive care unit (PICU), a 28-bed cardiac intensive care unit (CICU), and a 45-bed neonatal intensive care unit (NICU).

The results of continuous EEGs were recorded digitally using 21 scalp electrodes, affixed with paste or collodion adhesive, with a bedside computer networked to a central EEG server. There were no constraints placed on the number of cases placed on EEG at any one time. EEG technician coverage was available 24 h per day. EEG studies were reviewed by the attending neurophysiologist at least 3 times per 24 h but typically every 2–4 h or as necessary when seizures were active. The duration of monitoring was determined by the attending physician. Often, the duration would be between 24 and 48 h according to the common practice, but sometimes the duration would be much longer.

### Participants

The retrospective population consisted of all children who underwent cEEG from June 2010 to August 2013. This patient population was of very high acuity [for instance, the PICU was in the top 25% of participants in the Virtual Pediatric Systems database (16)]. To account for possible selection bias (as discussed in more detail later), we also compared this population with a more representative control sample [utilizing internal data from Children's Virtual Pediatric Systems, LLC (VPS) (16)] consisting of all PICU patients with neurological conditions at the Egleston campus of Children's from December 2010 to May 2014. Since most children in the control sample had not been monitored, this sample did not provide information on seizures, but it did contain conditions and age. Recorded conditions and ages then enabled a comparison of the control and monitored samples.

### Objectives

The primary objective was to identify subgroups of pediatric patients with high incidence of seizures. We defined such subgroups based on the indication for monitoring (also referred to as “diagnosis” in the following). New monitoring cases were identified from the neurophysiology database within 5 days from the start of the cEEG, on average within 24 h. The indication for the cEEG was abstracted from the chart at the time the study was ordered. Indications and patient characteristics included previous seizures, witnessed definite acute seizures, age, location, concerns of altered mental status, central nervous system (CNS) infection, hypoxic-ischemic encephalopathy (HIE), cerebrovascular accident (CVA), intracranial hemorrhage (ICH), acute trauma, toxic/metabolic encephalopathy, tumor, congenital malformation, spells (acute behavioral or physiological changes), or other reasons. All indications were recorded as binary variables if they were cited as reasons for initiating cEEG monitoring. A patient could have multiple indications (e.g., ICH and trauma).

### Outcomes

The primary outcome of the study was the incidence of any seizure. In the following, we describe how this outcome was measured.

After monitoring was completed, the duration of monitoring and time to first seizure and last seizure were determined by chart review of the neurophysiology reports. These studies were read by several different EEG readers who were neurologists trained in formal Epilepsy and EEG fellowships and had 7–25 years of experience in reading continuous EEG monitoring. It was also determined if “spells” occurred of the same type as the initial concerns, and whether or not they were seizures. If seizures had clear clinical behavioral change which could be recognized at bedside or on video, they were recorded as (clinical, “convulsive”) “seizures,” otherwise as non-convulsive seizures. When electrographic seizures occupied 50% or more of the EEG for longer than 30 min, they were labeled as “frequently repetitive seizures,” in order to identify a subgroup with extremely frequent, persistent, non-convulsive seizures. For purposes of this study, “seizures” refers to all three of these types of events. Electrographic seizures were considered separate from periodic or other rhythmic patterns if they had durations longer than 10 s, and evolved in frequency or field (not amplitude alone). Inter-rater reliability in seizure recognition was not explicitly examined, but notes were kept regarding any disparities. These were unusual and typically minor.

All three types of seizures (clinical, non-convulsive, and frequently repetitive) counted as a seizure in the statistical models. Seizure incidence served as the outcome variable in the models that follow while neurological conditions described in Objectives jointly served as explanatory variables. These variables were recorded consistently, with no missing observations.

### Sample size estimation

This was a retrospective study and no prior sample size determination had been conducted.

### Statistical analysis

In the following sections, we describe the methods that we used for the age threshold selection, seizure risk modeling, and selection bias correction. We conducted the analyses using the statistical software R (17). Unless stated otherwise, we report the statistical significance at the level of α = 0.05.

#### Age threshold selection

The literature indicates that infants and older children substantially differ in observed seizure incidence (9, 13) which suggests that for each of the two groups, different risk factors may play a role. Since the exact age where such a risk transition would occur is unknown, we stratified patients as infants or older children according to their observed seizure incidence using a statistical approach suggested by Ross and Tasoulis (18). This methodology assumes that children below and above a certain threshold age have unknown but different seizure probabilities. Then, a value of a statistic based on Fisher's exact test (19) determines the most appropriate age threshold to separate younger and older children. After this stratification, we conducted remaining analyses on each age group separately.

#### Seizure risk modeling

We used multivariate logistic regression (20) to estimate the seizure risk. The outcome variable was the occurrence of any seizures during EEG monitoring, but we also tested the outcome variable being non-convulsive seizures only. The multivariate model is stratified by age and includes as predictors all diagnoses that occurred in a sufficient number of patients. We do not further reduce the number of predictors because our sample size is sufficient, which is considered a good practice when the purpose of the model is effect estimation (21). We include results from univariate models for each of the diagnoses. Goodness-of-fit of the multivariate model was tested using the Hosmer-Lemeshow test (19). Next, to understand the effect of multiple conditions, we tested if seizure risk was magnified for children with multiple neurological conditions. Furthermore, to assess sensitivity to the choice of the age threshold, we reran the analysis with thresholds of 12 and 24 months. Also, since the control sample is drawn from PICU only, we reran the analysis on the subset of PICU children to see if the results qualitatively change. Further, since neonates (children under 1 month) have been studied in the literature separately and may differ from other young children, we conducted additional analyses separately for children under 1 month and between one and 14 months. Finally, we also considered two alternative models where we also included as covariates (a) having any other, infrequent diagnoses or (b) the type of ICU.

#### Selection bias correction

The ICU physicians ordered monitoring at their discretion which inherently might have induced a selection bias in our sample. We would expect that children were more likely to receive monitoring if perceived as being at high risk. Therefore, the children in our sample may exhibit higher prevalence of seizures than the general PICU neurological population. To partially account for this possible bias, we compared the monitored children to the ones from the control sample. We evaluated the selection bias using two different approaches, the inverse probability weighted estimation (IPWE) and the second method by Huang et al. (22). Both methods are described in more detail in Appendix 1 in Supplementary Material.

## Results

The dataset contained 517 pediatric monitored patients. Among the 150 patients who experienced seizures during cEEG monitoring, 68 had exclusively non-convulsive seizures. Because cerebrovascular accident, congenital malformation, toxic/metabolic encephalopathy, and tumor were each recorded in <10 patients in the younger age group, we excluded these conditions from the main model but kept them grouped within “Other diagnosis” in one of the supplementary models.

The age threshold selection yielded 14 months as the age cutoff. Of 517 children, 215 were 14 months or younger and 302 of them were older than 14 months, with median age of 24 months (IQR = 3 months−9 years). Most of the monitored children were in PICU (136 younger than 14 months and 287 older than 14 months). The monitoring duration was <12 h in 9% (*n* = 49) of patients, 12–24 h in 26% (*n* = 134), 24–48 in 31% (*n* = 162), 48–72 in 13% (*n* = 67) and more than 72 in 20% (*n* = 105). Among the children with seizures, the first seizure (after the start of monitoring) was observed in 57% within the first hour, in 22% within 1–6 h, in 7% within 6–12 h, in 6% within 12–24 h, and in 7% in more than 24 h. The first seizure time was missing in 15 children. Further summary statistics are provided in Table 1. Detailed breakdowns by departments (PICU/NICU/CICU) are available in Table 2. Six children not listed in the table were in Technology-dependent ICU (a lower acuity pediatric ward) and not included in the breakdown.

**Table 1:** The characteristics of monitored children (included are all children receiving cEEG during the study period; stratified by age group: younger or older than 14 months).

	**Age group**		
	**All**	**Younger than 14m**	**Older than 14m**
Total (*N* = 517)	517	215	302
**SEIZURES DURING MONITORING**, ***n*** **(%)**			
Convulsive seizures	80 (15)	48 (22)	32 (11)
Nonconvulsive seizures	116 (22)	75 (35)	41 (14)
Frequently repetitive seizures	68 (13)	46 (21)	22 (7)
Any seizures or status^*^	150 (29)	97 (45)	53 (18)
No seizures/status	367 (71)	118 (55)	249 (82)
**INDICATIONS FOR MONITORING**, ***n*** **(%)**			
Prior seizures/epilepsy	206 (40)	69 (32)	137 (45)
Intracranial hemorrhage (ICH)	77 (15)	47 (22)	30 (10)
Hypoxic-ischemic encephalopathy (HIE)	90 (17)	48 (22)	42 (14)
Head trauma	53 (10)	24 (11)	29 (10)
Infection (encephalitis or meningitis)	36 (7)	15 (7)	21 (7)
Coma or obtundation	98 (19)	17 (8)	81 (27)
Spells	170 (33)	93 (43)	77 (25)
Cerebrovascular accident	15 (3)	5 (2)	10 (3)
Congenital malformation	9 (2)	6 (3)	3 (1)
Toxic or metabolic encephalopathy	32 (6)	8 (4)	24 (8)
Tumor	27 (5)	3 (1)	24 (8)
Monitoring time (h), median (IQR)^**^	32 (20–64)	38 (22–71)	29 (19–53)
Time to first seizure (h), median (IQR)^***^	0.5 (0.2–4.3)	0.5 (0.0–4.3)	0.5 (0.0–4.2)

*IQR, Interquartile range in the format (1st quartile−3rd quartile)*.

* *Including convulsive seizures, non-convulsive seizures, and non-convulsive status epilepticus. Some patients may have two or three of these (e.g., both convulsive and non-convulsive seizures)*.

** *Monitoring time was unavailable for 31 children*.

*** *Among children with seizures. Time to first seizure was unavailable for 15 children*.

**Table 2:** The characteristics of monitored children, stratified by department (PICU/NICU/CICU) [excluded are 6 children monitored in Technology-dependent ICU (TICU)].

	**Department**		
	**PICU**	**NICU**	**CICU**
Total (*N* = 511)	423	45	43
Under 14m	136	45	31
**SEIZURES DURING MONITORING**, ***n*** **(%)**			
Convulsive seizures	64 (15)	9 (20)	6 (14)
Nonconvulsive seizures	96 (23)	11 (24)	8 (19)
Frequently repetitive seizures	60 (14)	5 (11)	3 (7)
Any seizures or status^*^	122 (29)	16 (36)	10 (23)
No seizures/status	301 (81)	29 (64)	33 (77)
**INDICATION FOR MONITORING**, ***n*** **(%)**			
Prior seizures/epilepsy	188 (44)	10 (22)	6 (14)
Intracranial hemorrhage (ICH)	59 (14)	9 (20)	9 (21)
Hypoxic-ischemic encephalopathy (HIE)	67 (16)	7 (16)	16 (37)
Head trauma	52 (12)	1 (2)	0 (0)
Infection (encephalitis or meningitis)	33 (8)	1 (2)	1 (2)
Coma or obtundation	91 (22)	1 (2)	5 (12)
Spells	122 (29)	29 (64)	14 (33)
Cerebrovascular accident	13 (3)	0 (0)	2 (5)
Congenital malformation	6 (1)	1 (2)	1 (2)
Toxic or metabolic encephalopathy	30 (7)	2 (4)	0 (0)
Tumor	26 (6)	0 (0)	0 (0)
Monitoring time (h), median (IQR)^**^	35 (21–68)	25 (20–44)	22 (15–43)
Time to first seizure (h), median (IQR)^***^	0.6 (0.0–4.6)	0.5 (0.3–1.8)	0.2 (0.1–2.1)

*IQR, Interquartile range in the format (1st quartile−3rd quartile)*.

* *Including convulsive seizures, nonconvulsive seizures, and nonconvulsive status epilepticus. Some patients may have two or three of these (e.g., both convulsive and nonconvulsive seizures)*.

** *Monitoring time was unavailable for 25 children in PICU, 3 in NICU, and 2 in CICU*.

*** *Among children with seizures. Time to first seizure was unavailable for 13 children in PICU and one in NICU*.

### Seizure risk

For children over 14 months, only previous seizures were associated in the multivariate model with significantly higher seizure risk (*p* < 0.001). Therefore, we focus on results for children under 14 months. Estimates and confidence intervals from the multivariate logistic regression model applied to children under 14 are presented in Table 3A. In this model, ICH, HIE, and infection were significantly associated with the increased risk of seizures. The Hosmer-Lemeshow test did not reveal a statistically significant lack of fit (*p* = 0.75), which suggested that our model is well calibrated. After refitting the model for children under 12 or under 24 months, ICH, HIE, and infection remained statistically significant, even though previous seizures were also significant at α = 0.05 level in both cases (*p* = 0.02 and 0.05, respectively). Similarly, the model counting only non-convulsive seizures resulted in the same set of significant predictors. Having multiple neurological conditions was associated with a higher seizure risk in children under 14 months [unadjusted odds ratio = 3.5; confidence interval (2.0, 6.3)]. The results were qualitatively similar when we reran the analysis on the subsample of PICU children. When the model was run separately for children under 1 month (*N* = 86) and the ones between one and 14 months (*N* = 129), the previously significant regression coefficients retained the same direction in both subsets. In the model that included a covariate for “Other diagnoses,” this covariate was not statistically significant. The model that controlled for the ICU type exhibited a lower risk for children in CICU, but the results for specific diagnoses were not substantially impacted (HIE, ICH, and infection remained statistically significant with similar point estimates). Detailed results for the last two models can be found in Table 3B.

**Table 3A:** Logistic regression of risk factors associated with any seizures among children under 14 months.

**Factor**	**Univariate analysis**		**Multivariate analysis**	
	**Odds ratio (95% CI)**	***p*-value**	**Odds ratio (95% CI)**	***p*-value**
Coma/obt.	1.41 (0.52, 3.89)	0.50	1.32 (0.43, 4.11)	0.62
Prior seizure	1.81 (1.02, 3.24)	0.04^*^	1.86 (0.94, 3.74)	0.08
Infection	2.60 (0.89, 8.59)	0.09	3.93 (1.26, 13.74)	0.02^*^
ICH	3.38 (1.73, 6.88)	<0.001^***^	3.05 (1.42, 6.74)	0.005^**^
HIE	2.79 (1.45, 5.54)	0.003^**^	2.95 (1.37, 6.60)	0.007^**^
Trauma	4.25 (1.70, 12.17)	0.003^**^	2.10 (0.70, 6.89)	0.20
Spells	0.39 (0.22, 0.68)	0.001^**^	0.80 (0.40, 1.64)	0.54
Multiple diagnoses	3.52 (2.01, 6.27)	<0.001^***^		

* p < 0.05;

** p < 0.01;

*** *p < 0.001. HIE, hypoxic-ischemic encephalopathy; ICH, intracranial hemorrhage*.

**Table 3B:** Logistic regression of risk factors associated with any seizures among children under 14 months: Supplementary multivariate models.

**Factor**	**Include Other Dx**		**Include Location (baseline** = **PICU)**	
	**Odds ratio (95% CI)**	***p*-value**	**Odds ratio (95% CI)**	***p*-value**
Coma/obt.	1.33 (0.43, 4.19)	0.62	1.20 (0.39, 3.71)	0.75
Prior seizure	1.88 (0.95, 3.78)	0.08	1.57 (0.77, 3.23)	0.21
Infection	4.16 (1.32, 14.70)	0.02	3.22 (1.01, 11.43)	0.05^*^
ICH	3.11 (1.44, 6.91)	0.004^**^	3.58 (1.61, 8.23)	0.002^**^
HIE	3.15 (1.44, 7.19)	0.005^**^	2.96 (1.36, 6.71)	0.007^**^
Trauma	2.20 (0.72, 7.35)	0.20	1.55 (0.49, 5.29)	0.47
Spells	0.85 (0.42, 1.76)	0.66	0.73 (0.35, 1.52)	0.40
Other Dx	1.53 (0.55, 4.21)	0.41		
CICU			0.38 (0.14, 0.98)	0.05^*^
NICU			0.71 (0.32, 1.55)	0.39
TICU			1.14 (0.05, 12.9)	0.92

* p < 0.05;

** *p < 0.01. HIE, hypoxic-ischemic encephalopathy; ICH, intracranial hemorrhage; PICU, pediatric ICU; CICU, cardiac ICU; NICU, neonatal ICU; TICU, technology-dependent ICU. Other Dx: One or more of cerebrovascular accident, toxic/metabolic encephalopathy, tumor, or congenital malformations*.

The results largely persisted in the selection-bias corrected models. Namely, the two correction methods that we used consistently pointed to a higher risk among children with ICH and HIE. Detailed results for the selection-bias corrected models can be found in the Appendix in Supplementary Material.

## Discussion

### Key results and interpretation

This study reveals that the high seizure risk previously reported in neonates might also apply to infants older than 1 month, namely children below 12–24 months (our best estimate of the threshold is 14 months). The critically-ill children under 14 months that have the diagnosis of HIE, ICH, or possibly meningitis or encephalitis are particularly at risk. These results complement risk factors identified in previous studies (2, 6–9, 13) including head trauma, recent prior seizures, acute presentation of epilepsy, and younger age. Head trauma did not appear as an independent predictor in our analysis possibly due to the fact that ICH, which is often associated with trauma, was included in the analysis. Compared to previous studies which were based on relatively smaller sample sizes (with 130 or fewer patients), our analysis with 517 patients is to our knowledge the largest single-institution study on pediatric cEEG monitoring. The only study with comparable size is a multi-center study (13) which included 550 patients. Compared with the study population in this multicenter study, our population is younger (median 24 vs. 36.5 months), and it includes a substantial number of children with acute structural neurological diagnoses such as HIE, ICH, CNS infection, and traumatic brain injury (48 vs. 24% in the multicenter study which may also suggest on average more severe patient population in our study). This allowed us to study the younger age group separately and uncover HIE, ICH, and CNS infection as risk factors for younger children, which were not found in the multicenter study.

Our dataset shows a very high rate of seizures (45%) among younger monitored patients (under 14 months). These numbers contrast with the seizure rate of 18% observed in the monitored children over 14 months which is on par with the rates observed in adult ICU units [(23), Figure 5.1]. Even in the entire neurological PICU population under 14 months, our estimates indicate a seizure rate around 30%. Higher seizure rates for younger children have been previously observed in other studies (2, 9, 13). Our findings indicate that distinguishing younger and older age groups of children in monitoring decisions is essential.

Based on our results, monitoring all children 14 months of age or younger with ICH or HIE may be warranted. In our population, this rule would capture 53% of monitored patients with any diagnosis under 14 months for which seizures were detected. In other words, its sensitivity is 53%. The rule would also have the positive predictive value of 62% in the monitored sample. Of course, whether these results generalize to unmonitored children is unclear. Assuming so, in our control sample, just 33% (45 out of 136) of children under 14 months with ICH or HIE received any EEG monitoring. Among the other 91 unmonitored children, our estimates suggest that as many as 53 may actually have suffered from seizures and most of these were not clinically detected. Although more extensive monitoring may be warranted, it would require additional resources. In fact, if all these children were monitored, the total volume of EEG monitoring in PICU would have been 16% higher (587 monitored children compared to the actual 496 monitored children). We acknowledge that there may be other reasons not to initiate monitoring in some high risk patients. For example, clinical setting and lack of brain function on exam with a dismal prognosis for survival may justify withdrawal of care without ICU EEG.

We concur with Abend et al. (13) that a multicenter prospective screening study of all children in PICU for specified cEEG indications is desirable. Until then, our study provides a better understanding of the younger patient population, which was relatively pauce in prior studies.

### Limitations

The main limitation of our study is the retrospective design. Also, our study population consisted of children from only one hospital, so the acuity and diagnoses of patients may not be fully representative of general pediatric ICU population with neurological conditions. The next limitation is the lack of guidelines and recommendations regarding patient selection for cEEG monitoring. Although an institutional brain trauma pathway provides recommendations for antiepileptic therapy and suggests cEEG monitoring, even for these patients, the ICU physician had the ultimate authority in the decision whether to monitor. In terms of anticonvulsants, while a detailed analysis thereof was not within the scope of this study, the neurologists and intensivists were all operating within an accepted treatment protocol, so treatment approaches would be similar for all patients. Factors that influence the decision to monitor might include ICU, provider, diagnosis, or age, and could vary over time with experience with which cases are high or low risk. Furthermore, the data did not include any scores measuring the severity of illness. We attempted to address some of these selection bias limitations (in particular, the *selection on observables*) by using a correction method. Finally, even though we observe a markedly higher seizure rate in infants (45% in monitored children under 14 months compared to 18% in older children), the dearth of children between 12 and 20 months in our cohort does not allow us to determine how steep the drop in the seizure rate is as a function of age.

### Future research

Our current work raises new questions that should be addressed by future studies. Other than previous seizures, we were unable to detect any significant risk factors in older children. For comparison, in adults certain neurological conditions are associated with higher risk, but this effect may be less salient in young children (24). Guided by these insights, we conducted a simple power analysis which indicated that our study would reliably detect conditions with associated seizure risk above 50%, if there were any. However, to detect the effect size corresponding to a risk factor associated with seizure risk of 30% (as opposed to the standard 18% baseline risk), we would need as many as 1,500 patients to ensure the statistical power of 0.8 (a conventionally-assumed value in the study design). Due to the small number of patients, we also did not explore the effect of certain conditions listed in the Materials and Methods section. Therefore, these conditions and their impact on seizure risk also require further investigation.

## Conclusions

In this study, which is the largest single-center retrospective review of pediatric patients receiving cEEG monitoring to-date, we observe an extremely high incidence of seizures in critically-ill children with neurological conditions under 14 months. In these children, the following factors are associated with an increased risk of seizures: intracranial hemorrhage, hypoxic-ischemic encephalopathy, and meningitis or encephalitis. Under the current practice, many of these children who do not undergo cEEG monitoring may suffer from non-convulsive seizures.

The study highlights the extraordinary vulnerability of the infant brain to the occurrence of acute symptomatic seizures. For providers who care for young patients, this special vulnerability should be reflected in their vigilance to and management of these patients and their problems.

## Ethics statement

The Institutional Review Board of Children's Healthcare of Atlanta approved this study (IRB protocol number 13-009). For this type of study, formal human participant consent was not required.

## Author contributions

JV collected and analyzed data and wrote and edited the manuscript. MJ and QL performed data collection and analysis. TA and PK supervised and mentored JV, MJ, and QL assisted with data analysis and edited and revised the manuscript. JS was involved in study design, and manuscript revision. LO initiated the study, collected the data, and supervised all aspects of the study and manuscript preparation. AV is the primary mentor and coordinator for this study, provided oversight for all participants and reviewed and revised the manuscript.

### Conflict of interest statement

The authors declare that the research was conducted in the absence of any commercial or financial relationships that could be construed as a potential conflict of interest.

## Abbreviations

cEEG: continuous electroencephalography
Children's: Children's Healthcare of Atlanta
CICU: cardiac intensive care unit
CNS: central nervous system
EEG: electroencephalography
HIE: hypoxic-ischemic encephalopathy
ICH: intracranial hemorrhage
ICU: intensive care unit
IPWE: inverse probability weighted estimation
IQR: inter-quartile range
NICU: neonatal intensive care unit
OR: odds ratio
PICU: pediatric intensive care unit
VPS: Virtual Pediatric Systems, LLC.
