# Supplementary material for: Risk Factors for Seizures Among Young Children Monitored With Continuous Electroencephalography in Intensive Care Unit: A Retrospective Study
Source: Front Pediatr. 2018 Oct 15;6:303. doi: 10.3389/fped.2018.00303 (PMC6196272; doi:10.3389/fped.2018.00303)
Supplement: Supplementary file 1 [file Data_Sheet_1.docx]

Appendix 1

Risk factors for seizures among young children in ICU

# Appendix 1A: Power analysis

We used the method described in [Hsieh et al. ^1^](#_ENREF_1) First, to understand why past studies failed to detect these predictors, we estimated the sample sizes that would have been needed to detect the effects seen in younger children. Second, we estimated the sample sizes that would be needed in older children if the effect sizes were of similar sizes as the ones typically seen in adults. These numbers may guide the design of future studies.

| Condition | Required sample size |
| --- | --- |
| CNS infection | 194 |
| ICH | 115 |
| HIE | 121 |

Table 1: Sample sizes required in young children population to detect significance, assuming power 50%, and significance level 95%, and effect sizes as observed in our study.

| Condition | Required sample size for P=0.3 | Required sample size for P=0.5 |
| --- | --- | --- |
| Coma/Obtundation | 492 | 73 |
| CNS infection | 1520 | 226 |
| ICH | 1272 | 189 |
| HIE | 883 | 131 |
| Trauma | 1327 | 197 |
| Known seizures | 444 | 65 |
| Spells | 584 | 86 |

Table 2: Sample sizes required in older children population, assuming power 80%, significance level 95%, and effect sizes as typically observed in adults (corresponds to prevalence of seizures P=30% under the observed condition) or higher ones, as observed in younger children in our study (seizure prevalence P=50%), both assuming baseline prevalence of seizures being 18%.

# Appendix 1B: Selection bias correction

We evaluated the selection bias using two different approaches, which we now briefly outline (details for both approaches are in the appendix). The first approach, the inverse probability weighted estimation (IPWE) fits a model based on the original monitored sample, but reweighs this sample to put larger weight on children whose condition is underrepresented (with respect to the control sample) and smaller weight for the children whose condition is overrepresented. The second method, by Huang et al. is also based on sample weighting, but it additionally precludes “excessive” over- or under-sampling. We note that these methods are applicable even though the monitored sample and the control sample partially overlap. After the selection bias correction, we compared the coefficients and statistical significance for the risk factors in the refitted model to the ones in the original regression. Finally, using our model, we estimated the seizure risk in the entire neurological PICU population rather than just within the monitored sample. Because the original model was based on both children monitored in PICU as well as other ICUs, we also compared the original model applied to the PICU-only population.

We corrected the selection bias using the inverse probability weighted estimation (IPWE) approach[^2^](#_ENREF_2) and the approach from [Huang et al. ^3^](#_ENREF_3) Both methods refit the logistic regression model but now with the weights $s_{i}$ given to the i-th observation (where $s_{i}=1$ in the original, uncorrected model). In IPWE, $s_{i}$ are computed as the predicted probabilities of being prescribed an EEG monitoring given the patient’s conditions. The parameters to predict the probabilities are estimated using a logistic regression on the control sample (the outcome variable: being prescribed EEG, the predictors: conditions) and then used to estimate $s_{i}$ for every patient in the monitored sample. In contrast, [Huang et al. ^3^](#_ENREF_3) argue that such a method might be very fragile when probabilities are not estimated precisely; they suggest that their approach is more robust. Simply speaking, they choose $s_{i}$ in a way that the monitored sample and the control sample “look similar” in terms of the predictor variables. Their approach eventually computes the weights $s_{i}$ (we denote the vector of all of them as $\boldsymbol{s}$) as a solution of the following minimization problem (as adapted for our problem):

$\text{minimiz}\text{e}_{\text{s}} \frac{1}{2} \boldsymbol{s}^{T}\boldsymbol{x}\boldsymbol{x}^{T}\boldsymbol{s}-\frac{N}{N^{'}} \left( \sum_{j=1}^{N^{'}} \boldsymbol{x}x_{j}' \right)^{T}\boldsymbol{s}\boldsymbol{,}\text{subject to }s_{i}\in\left[ 0,B \right]\text{and} \left| \frac{1}{N}\sum_{i=1}^{N} s_{i}-1 \right|\leq\frac{B}{\surd N}$ **,**

where $\boldsymbol{x}, \boldsymbol{x}'$ are the matrices of the monitored, respectively control samples (${e.g. x}_{ij}=1$if the i-th patient in the monitored sample has the j-th condition, and 0 if they do not have it), N, N’ are the sample sizes of the monitored, respectively control sample, and B is a parameter to be chosen. Based on the arguments given in the article, we experimented with different sizes of B=1.5, 3, 5, 10, and eventually chose the weights with B=1.5 to prevent over-weighting. Nevertheless, reweighting decreased the effective sample size substantially which in turn resulted in less stable estimates. We excluded infection because it matched poorly to the control sample. The equation above is a quadratic optimization problem and was solved using IBM ILOG CPLEX V12.6.0. The results in Table 3 for this methods are somewhat suggestive of higher risk due to ICH and HIE, but possibly not entirely reliable.

In view of the significant findings in older children, we assessed the selection bias in younger children under 14 months. After the selection bias correction with the IPWE method, ICH (p=0.07, OR: 2.3) and HIE (p<0.01, OR: 3.1) remained significant risk factors. Huang’s method provided consistent but less reliable estimates. In contrast, CNS infection was not significantly associated with seizures after the selection bias correction. The selection-bias-corrected model predicted the seizure rate as 32% in the entire neurological PICU sample which was predicted as 30% in the original model.

| Factor | Method (OR, 95% CI) | |
| --- | --- | --- |
|  | IPWE | Huang |
| Coma/obt. | 1.38 (0.34, 5.58) | 0.22 (0.02, 2.00) |
| Prior seizure | 1.94 (0.99, 3.80) | 0.50 (0.04, 6.48) |
| Infection | 1.92 (0.61, 6.10) | NA |
| ICH | 2.27 (0.93, 5.54) | 56.80 (1.09, 2965.92) |
| HIE | 3.07 (1.34, 7.06) | 5.28 (0.58, 48.07) |
| Trauma | 1.92 (0.60, 6.18) | 0.05 (0.00,1.52) |

Table 3: Multiple logistic regression after the selection bias adjustment using two procedures. Spells were excluded in both cases because the control dataset did not record them. Also, infection was excluded for the Huang’s method because the match between the control and monitored sample was not good enough.
OR, odds ratio; CI, confidence interval; HIE, hypoxic-ischemic encephalopathy; ICH, intracranial hemorrhage

1. Hsieh F, Bloch D, Larsen M. A simple method of sample size calculation for linear and logistic regression. *Statistics in Medicine* 1998;17:1623-1634.

2. Wooldridge J. Econometric analysis of cross section and panel data. MIT Press; 2010.

3. Huang J, Gretton A, Borgwardt KM, et al. Correcting sample selection bias by unlabeled data. *Advances in neural information processing systems* 2006:601-608.
